# The Tumor-Log Odds of Positive Lymph Nodes-Metastasis Staging System, a Promising New Staging System for Gastric Cancer after D2 Resection in China

**DOI:** 10.1371/journal.pone.0031736

**Published:** 2012-02-14

**Authors:** Miao-zhen Qiu, Hui-juan Qiu, Zhi-qiang Wang, Chao Ren, De-shen Wang, Dong-sheng Zhang, Hui-yan Luo, Yu-hong Li, Rui-hua Xu

**Affiliations:** 1 State Key Laboratory of Oncology in South China, Guangzhou, China; 2 Department of Medical Oncology, Sun Yat-Sen University Cancer Center, Guangzhou, China; 3 Department of Traditional Chinese Medicine, Sun Yat-Sen University Cancer Center, Guangzhou, China; The Chinese University of Hong Kong, Hong Kong

## Abstract

**Background:**

In this study, we established a hypothetical tumor-lodds-metastasis (TLM) and tumor-ratio-metastasis (TRM) staging system. Moreover, we compared them with the 7^th^ edition of American Joint Committee on Cancer tumor-nodes-metastasis (AJCC TNM) staging system in gastric cancer patients after D2 resection.

**Methods:**

A total of 1000 gastric carcinoma patients receiving treatment in our center were selected for the analysis. Finally, 730 patients who received D2 resection were retrospectively studied. Patients were staged using the TLM, TRM and the 7^th^ edition AJCC TNM system. Survival analysis was performed with a Cox regression model. We used two parameters to compare the TNM, TRM and TLM staging system, the −2log likelihood and the hazard ratio.

**Results:**

The cut points of lymph node ratio (LNR) were set as 0, 0–0.3, 0.3–0.6, 0.6–1.0. And for the log odds of positive lymph nodes (LODDS), the cut points were established as≤−0.5, −0.5-0, 0-0.5, >0.5. There were significant differences in survival among patients in different LODDS classifications for each pN or LNR groups. When stratified by the LODDS classifications, the prognosis was highly homologous between those in the according pN or LNR classifications. Multivariate analysis showed that TLM staging system was better than the TRM or TNM system for the prognostic evaluation.

**Conclusions:**

The TLM system was superior to the TRM or TNM system for prognostic assessment of gastric adenocarcinoma patients after D2 resection.

## Introduction

Approximately one million people are diagnosed each year with gastric cancer, making it the fourth most common cancer and the second leading cause of cancer related death worldwide with an estimated 800,000 deaths caused by the disease [1]. The incidence of gastric cancer varies widely according to geographic region and is particularly common in Asia [2]. Until now the prognosis for gastric adenocarcinoma patients stays poor and Tumor-Node-Metastasis (TNM) staging system has been proved to be a prognostic factor which can effectively predict the prognosis of gastric adenocarcinoma patients [3]. From January 1, 2010 on, the most recent revision of American Joint Committee on Cancer (AJCC) TNM stage for carcinoma of gastric (the 7^th^ edition) was put into use [4]. Our previous study has shown that the 7^th^ edition of AJCC TNM staging system was more reasonable compared with the AJCC 6^th^ system in predicting the survival of gastric cancer patients to a certain degree [5]. However, some authors pointed out that the value of the latest number-based pN classification in the AJCC TNM staging system was affected by the number of lymph nodes retrieved [6]–[13]. A new ratio-based lymph nodes system (rN) has been proposed, which was defined as the ratio of the metastatic lymph nodes and the total number of retrieved lymph nodes after the resection. Recently, some studies has indicated that the TRM (Tumor-Ratio-Metastasis) staging system can be an alternative to the traditional TNM staging system [14]. However, some authors concerned that almost half of the Asian patients would not benefit from the ratio-based classification system since the definition of the rN0 classification was congruent with the pN0 classification [13].

Log odds of positive lymph nodes (LODDS), is defined as the log of the ratio between numbers of positive lymph nodes and the numbers of negative lymph nodes. To avoid singularity, 0.5 is usually added to both the numbers of positive lymph nodes and negative lymph nodes, log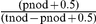, in which the pnod is the number of positive lymph nodes and tnod means the total number of lymph nodes retrieved [15]. Sun et al. studied 2,547 gastric cancer patients and concluded that the LODDS system was more reliable than the Union Internationale Contre le Cancer (UICC) and AJCC pN system and the rN system for prognostic assessment [13]. Till now, there is no study focus on the prognostic significance of the tumor-lodds-metastasis (TLM) stage system for gastric cancer patients after D2 resection. The aim of our study is to compare the TLM, tumor-ratio-metastasis (TRM) and the 7th AJCC TNM staging system in prognostic assessment for carcinoma of the gastric after D2 resection in China.

## Results

### Patient demographics

The median age of the 730 patients was 60 years (range 24–83 years). Among them, 522 were male and 208 were female. The overall 5-year survival for the whole group of patients was 55.4%, with median survival of 78.0 months. The median follow-up for the entire cohort was 48.0 months (range 3.0–175.0 months). The characteristics of the 730 gastric adenocarcinoma patients and the effect of clinical features on survival were summarized in Table 1. The total number of dissected lymph nodes was 12374, with an average of 17.0±11.4 (means±s.d.) dissected nodes per case (median 16.0, range 0–72). The mean number of metastatic nodes was 7.8±5.0 (median 4, range 0–70) in the overall series and 9.7±7.6 (median 7, range 1–70) in lymph nodes positive patients. The number of excised lymph nodes was less than 15 in 21.6% of patients who received resection.

**Table 1:** Demographics and univariate survival analysis results of the 730 gastric carcinoma patients.

Factors	Numbers	5 years OS (%)	*P* value
Gender			
Male	522	55.2	
female	208	56.0	0.544
Age median 60			
<60	386	60.0	
≥60	344	50.2	0.004
Tumor size			
≤5 cm	470	61.5	
>5 cm	260	44.7	<0.001
Anemia			
Yes	127	60.6	
No	309	70.1	0.038
Location of tumor			
Proximal	304	46.4	
distal	426	58.3	<0.001
Degree of differentiation			
Well+Moderate	200	54.1	
Poor+signet ring cell	530	45.8	0.007
Total number of LN retrieved			
<15	158	47.5	
≥15	572	60.2	<0.001
The 7^th^ T stage (AJCC)			
T1	144	91.8	
T2	179	78.2	
T3	133	57.8	
T4	274	49.4	<0.001
The 7^th^ N stage (AJCC)			
N0	267	72.1	
N1	113	63.7	
N2	168	53.9	
N3	182	26.8	<0.001
The R stage (LN)			
R0	267	72.1	
R1	195	65.6	
R2	143	30.3	
R3	125	13.0	<0.001
The L stage (LN)			
LODDS1	305	71.2	
LODDS2	174	47.9	
LODDS3	142	25.9	
LODDS4	109	14.8	<0.001
The 7^th^ TNM stage (AJCC)			
IA	31	92.3	
IB	35	87.2	
IIA	32	74.2	
IIB	220	71.3	
IIIA	85	56.5	
IIIB	145	46.7	
IIIC	182	26.3	<0.001
The TRM stage			
IA	31	92.3	
IB	39	88.7	
IIA	40	83.9	
IIB	212	68.7	
IIIA	135	60.7	
IIIB	142	35.7	
IIIC	131	20.5	<0.001
The TLM stage			
IA	40	93.8	
IB	55	85.9	
IIA	35	78.3	
IIB	242	65.2	
IIIA	108	52.3	
IIIB	136	30.1	
IIIC	114	12.4	<0.001

Abbreviations: AJCC, American Joint Committee on Cancer; TNM, Tumor-Node-Metastasis; TRM, Tumor-Ratio-Metastasis; TLM, Tumor-Lodds-Metastasis.

### The classification of rN and LODDS intervals

**Figure 1:**
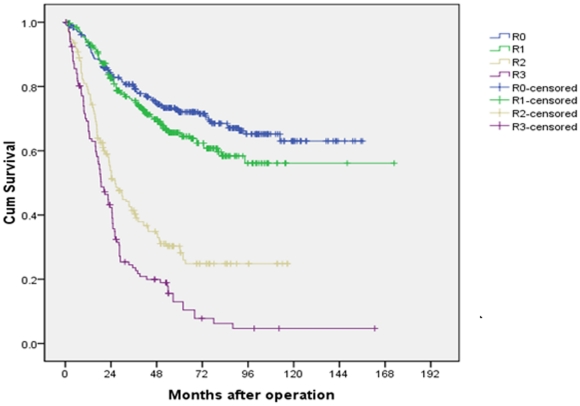
Survival curves of gastric cancer patients with D2 resection according to rN stage.

Table 2 listed the patient numbers and the 5-year survival rates of different groups according to the value of rN with an interval of 0.1 (ranging from 0 to 1.0). As shown, 4 groups were identified by combining patients with similar prognosis. Accordingly, a novel N classification, rN classification was established: R0 (rN = 0), R1 (0<rN≤0.3), R2 (0.3<rN≤0.6), R3 (0.6<rN≤1.0). The 5-year survival rates of R0, R1, R2 and R3 patients were 72.1%, 65.6%, 30.3% and 13.0%, respectively (P<0.001, Figure 1).

**Table 2:** Overall survival rates of gastric cancer patients with D2 resection according to the value of lymph nodes ratio (rN) with the interval of 0.1 (ranging from 0 to 1).

	No.	5-YSR(%)	P^a^
rN = 0	267	72.1	0.039
0<rN≤0.1	66	68.0	0.897
0.1<rN≤0.2	70	67.3	0.921
0.2<rN≤0.3	59	67.5	0.007
0.3<rN≤0.4	62	40.2	0.373
0.4<rN≤0.5	52	33.9	0.677
0.5<rN≤0.6	29	32.9	0.021
0.6<rN≤0.7	33	24.4	0.418
0.7<rN≤0.8	39	21.3	0.092
0.8<rN≤0.9	22	12.7	0.597
0.9<rN≤1.0	31	10.2	

5-YSR, 5-year survival rate.

a Compared between adjacent groups.

**Figure 2:**
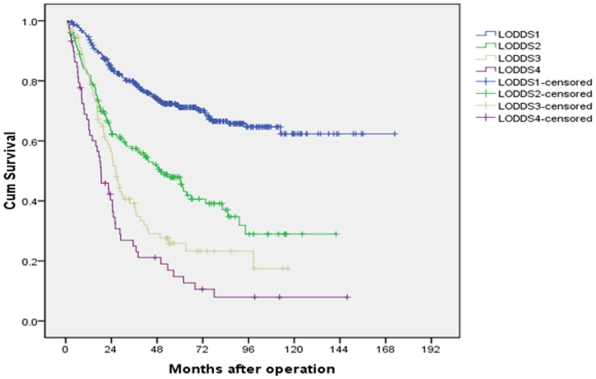
Survival curves of gastric cancer patients with D2 resection according to LODDS stage.

The value of LODDS ranged from −2.05 to 1.93. Table 3 listed the patient numbers and the 5-year survival rates of different groups according to the value of LODDS with an interval of 0.5. Since only three patients with a LODDS smaller than −2.00, we combined patients in the group LODDS≤−2.00 and patients in the group −2.00<LODDS≤−1.50 together. As shown, we identified 4 groups by combining patients with similar prognosis. Another novel N classification, LODDS classification was then established: LODDS1 (LODDS≤−0.5), LODDS2 (0.5<LODDS≤0), LODDS3 (0<LODDS≤0.5), LODDS4 (0.5<LODDS). The 5-year survival rates of LODDS1, LODDS2, LODDS3 and LODDS4 patients were 71.2%, 47.9%, 25.9% and 14.8%, respectively (P<0.001, Figure 2).

**Table 3:** Overall survival rates of gastric cancer patients with D2 resection according to the value of LODDS with the interval of 0.5 (ranging from −2.05 to 1.93).

	No.	5-YSR(%)	P^a^
LODDS≤−1.5	96	75.7	0.418
−1.5<LODDS≤−1.0	123	73.4	0.270
−1.0<LODDS≤−0.5	86	65.3	<0.001
−0.5<LODDS≤0	174	47.9	0.009
0<LODDS≤0.5	142	25.9	0.005
0.5<LODDS≤1.0	48	12.2	0.342
1.0<LODDS≤1.5	28	17.0	0.437
LODDS>1.5	33	11.0	

5-YSR, 5-year survival rate.

a Compared between adjacent groups.

**Figure 3:**
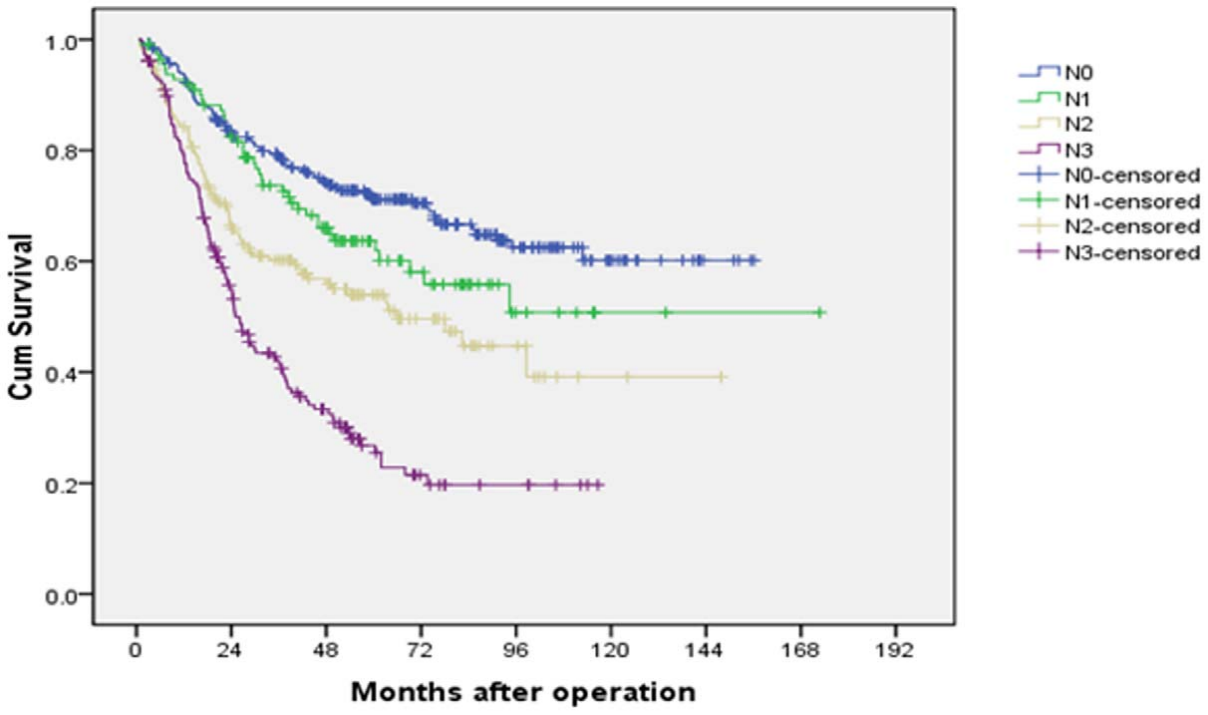
Survival curves of gastric cancer patients with D2 resection according to AJCC 7^th^ N stage.

The 5-year survival rates of N0, N1, N2 and N3 (AJCC N classification) patients were 72.1%, 63.7%, 53.9% and 26.8%, respectively (P<0.001, Figure 3).

The Kaplan-Meier plots shown a good discriminatory ability among each group in these three N classifications.

Table 4 listed the 5-year survival rates of patients with different pN and rN classifications, stratified by LODDS. As shown, for patients in each of the pN or rN classification, significant differences in survival could always be observed among patients in different LODDS classification. For patients in each LODDS classification, prognosis was highly homologous between those in different pN or rN classifications. These results indicated that the LODDS classification is superior to the pN and rN classifications for prognostic assessment.

**Table 4:** Overall survival rates with different pN and rN classifications stratified by the LODDS staging system.

	LODDS1		LODDS2		LODDS3		LODDS4		*P* a
	No	5-YSR(%)	No.	5-YSR(%)	No.	5-YSR(%)	No.	5-YSR(%)	
N stage									
N0	209	71.1	48	61.8	10	42.6	-	-	0.035
N1	51	68.0	39	55.2	23	33.3	-	-	0.04
N2	37	77.2	59	49.2	40	29.1	32	18.2	<0.001
N3	8	75.0	28	38.8	69	24.7	77	13.6	0.005
*P* b	0.796		0.396		0.872		0.892		
R stage									
R0	205	70.3	54	60.4	8	42.0	-	-	0.009
R1	100	71.3	85	51.6	10	29.4	-	-	0.026
R2	-	-	35	47.3	78	28.7	30	15.4	0.018
R3	-	-	-	-	46	26.3	79	14.8	0.003
*P* c	0.827		0.497		0.329		0.920		

Abbreviations: LODDS, Log Odds of Positive Lymph Nodes; No, number of patients; 5-YSRs, 5-year survival rate.

a Comparison of overall survival rates between different LODDS groups.

b Comparison of overall survival rates between different pN groups.

c Comparison of overall survival rates between different rN groups.

### Univariate and multivariate analyses of 5-year overall survival

**Figure 4:**
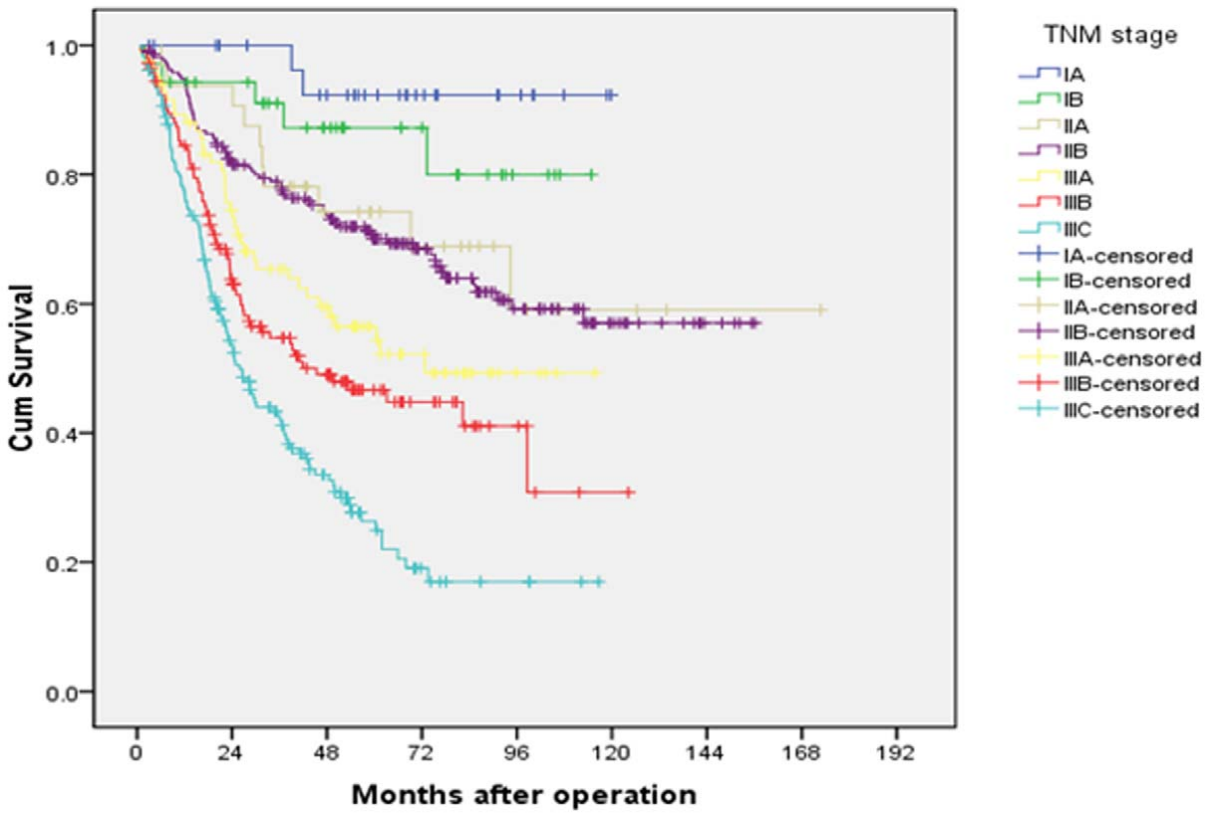
Survival curves of gastric cancer patients with D2 resection according to AJCC TNM staging system.

**Figure 5:**
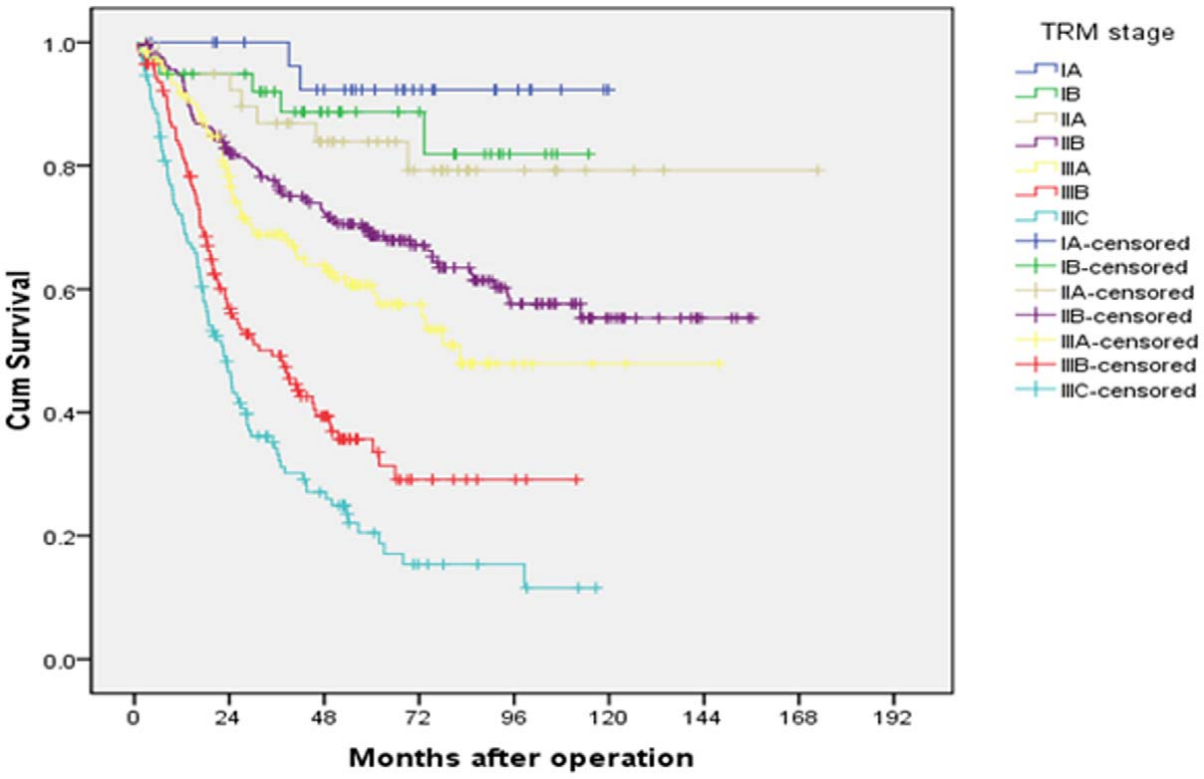
Survival curves of gastric cancer patients with D2 resection according to TRM staging system.

**Figure 6:**
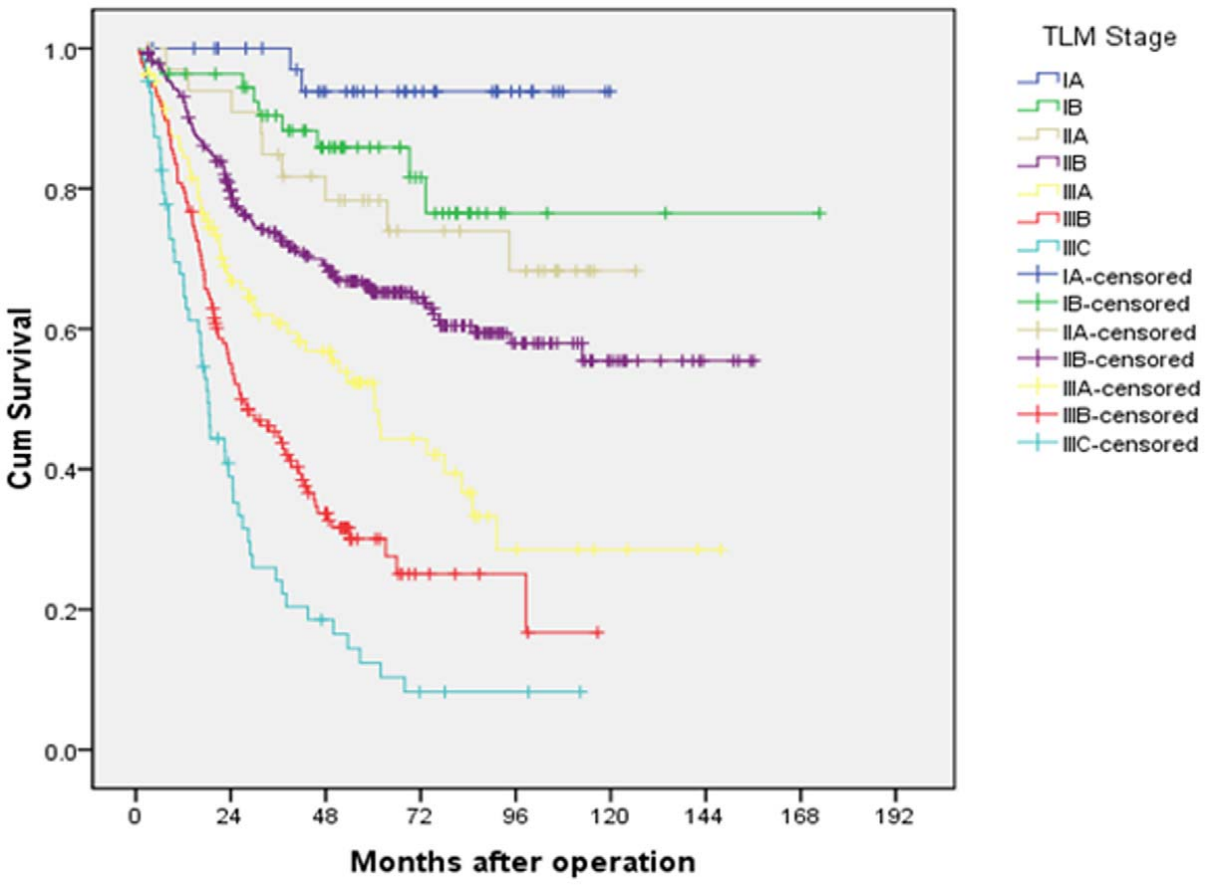
Survival curves of gastric cancer patients with D2 resection according to TLM staging system.

Both univariate and multivariate analyses were used to evaluate factors relating to 5-year overall survival. The items of age, tumor size, status of anemia, location of tumor, degree of differentiation, total number of lymph nodes retrieved, pT classification, pN classification, rN classification, LODDS and three staging systems were significantly related to 5-year overall survival (Table 1). In the AJCC 7^th^ TNM staging system the 5-year overall survival rates of patients from stage IA to stage IIIC were 92.3% vs 87.2% vs 74.2% vs 71.3% vs 56.5% vs 46.7% vs 26.3%, respectively (P<0.001, Figure 4). There was similar survival curves between stages IIA and IIB. While in the TRM and TLM staging systems, no overlapping survival curve was found in the seven subgroups (Figures 5 and 6). The 5-years survival rates of patients from stage IA to stage IIIC in the TRM staging system were 92.3% vs 88.7% vs 83.9% vs 68.7% vs 60.7% vs 35.7% vs 20.5% (P<0.001). In the TLM staging system, the survival rates were 93.8% vs 85.9% vs 78.3% vs 65.2% vs 52.3% vs 30.1% vs 12.4%, respectively (P<0.001).

For the multivariable regression analysis, we firstly set up a model including age, status of anemia, size of tumor, tumor location, degree of differentiation, total number of lymph nodes retrieved and AJCC 7th TNM staging system. Then we set up a second model which was identical to the first one except that the AJCC 7th TNM staging system was replaced by the TRM staging system. In the third model we used the TLM staging system to replace the TRM system. We used two parameters to compare the TNM, TRM and TLM staging system, the −2log likelihood and the hazard ratio (HR). The higher the HR, the better the system. While the smaller the −2log likelihood, the better the system. Though in the three multivariable regression analysis systems, TNM, TRM and TLM were all independent factors for the overall survival (P<0.001 for these three parameters, Table 5). We found that the −2log likelihood of these three staging system were 1393.437, 1386.707 and 1382.555 for the TNM, TRM and TLM staging system, respectively. While the HRs were 1.366, 1.463 and 1.504 for the TNM, TRM and TLM staging system, respectively. Therefore we considered the TLM system was superior to the TRM and TNM system (Table 5).

**Table 5:** Three steps multivariate analysis of overall survival in gastric carcinoma.

Factors	Characteristics		Multivariate Analysis 1			Multivariate Analysis 2			Multivariate Analysis 3		
	Unfavorable	Favorable	Hazard ratio	95%CI	*P* value	Hazard ratio	95%CI	*P* value	Hazard ratio	95%CI	*P* value
Age	≥60	<60	1.016	1.000–1.032	0.056	1.258	0.881–1.796	0.206	1.302	0.913–1.858	0.145
Anemia	Yes	No	1.509	0.975–2.335	0.065	1.692	1.099–2.606	0.017	1.710	1.110–2.635	0.015
Size	≥5 cm	<5 cm	1.512	1.059–2.158	0.023	1.542	1.082–2.198	0.017	1.544	1.084–2.200	0.016
Location	Proximal	Distal	0.730	0.497–1.073	0.109	0.742	0.509–1.081	0.120	0.753	0.515–1.100	0.142
Degree of differentiation	Poor+signet ring cell	Well+Moderate	0.599	0.379–0.946	0.028	0.588	0.375–0.924	0.021	0.569	0.362–0.895	0.015
Total number of LN retrieved	<15	≥15	0.702	0.478–1.032	0.072	0.865	0.600–1.246	0.437	1.395	0.668–1.395	0.850
AJCC 7^th^ TNM stage	III+IV	I+II	1.366	1.166–1.601	<0.001						
TRM stage	III+IV	I+II				1.463	1.286–1.664	<0.001			
TLM stage	III+IV	I+II							1.504	1.320–1.713	<0.001

Abbreviations: CI, confidence interval; LN, lymph node; AJCC, American Joint Committee on Cancer; TNM, Tumor-Node-Metastasis; TRM, Tumor-Ratio-Metastasis; TLM, Tumor-Lodds-Metastasis.

## Discussion

For decades, the involvement of regional lymph nodes with cancer in malignant diseases has been considered as one of the most important prognostic factors. Other information pertaining to the total numbers of lymph nodes and negative lymph nodes has become the focus of studies in these years [15]. LNR and LODDS were two new indices that have been considered important and promising recently. The superiority of LNR as a prognostic classification in various malignancies, including gastric cancer, compared to the pN classification which is basing on the absolute number of metastasis lymph nodes (MLN) in predicting prognosis of gastric cancer patients [10], [12], [14].

There is little data on the study of LODDS. Considering its unique statistical characteristic, LODDS has the potential to become a superior prognostic index. Our study shown that the LODDS classification was superior to the pN and rN classifications for prognostic assessment. In an analysis of the prognostic factors related to lymph nodes in 24,477 colon cancer patients extracted from the SEER database, Wang et al. [15] concluded that LODDS was a better prognostic factor than LNR. Vinh-Hung et al. [16] and Yildirm et al [17] both reached another conclusion that the estimated LODDS provided similar result to those with LNR basing on the analysis of node positive breast cancer patients. There were several reasons that made LODDS classification superior to the rN and pN classification. Sun et al. [13] proposed that it might because of its potential of discriminating patients with the same ratio of nodes metastasis but different survival. Wang et al. [14] considered that LODDS was a function of the number of negative lymph nodes, whereas LNR was a function of total number of lymph nodes. In our study, we compared the overall survival rates of patients in different pN, rN and LODDS classifications and we observed that all the three N classifications were all significant different in predicting the survival. Moreover, we found that the significant differences in survival could always be found for patients in each of the pN or rN classifications when stratifying by LODDS. However, prognosis was highly homologous for patients in each of the LODDS classifications when stratifying by the pN or rN classifications. It is one evidence showing that the superiority of LODDS over the LNR or the AJCC N stage in gastric cancer.

Wang et al. [14] analyzed 1343 cases of gastric cancer patients who underwent D2 resection and clasified the cut points of LNR as 0, 0–0.3, 0.3–0.6 and >0.6. They concluded that the TRM staging system may be considered as an alternative to the 7^th^ TNM system. While in some other reports the best cut points of LNR differed. In the study carried out by Bando et al. [18], it was 0, 0–0.1, 0.1–0.25 and ≥0.25. Sun et al. [13] analyzed 2547 cases of gastric cancer patients and classified the best cut points of LNR as 0, 1–0.2, 0.21–0.5 and >0.5. The intervals of N ratio classification were determined in our study by comparing the overall survival rates according to the rN with an initial interval of 0.1 and combing patients with similar prognosis. The intervals of LODDS were also determined by using the best cutoff approach and considering the patients' survival (log-rank statistic) with an initial interval of 0.5 as the dependent variable. According to this, in our manuscript, the cut points of lymph node ratio were set as 0, 0–0.3, 0.3–0.6, 0.6–1.0. And for the log odds of positive lymph nodes (LODDS), the cut points were established as≤−0.5, −0.5-0, 0-0.5, >0.5. Only 4 groups were identified by combining patients with similar prognosis which is comparible with the N classification in the AJCC 7^th^ staging system. While Sun et al. [13] established the LODDS classifications as ≤−1.5, −1.5<LODDS≤−1.0, −1.0<LODDS≤−0.5, −0.5<LODDS≤0 and >0.

Basing on the superiority of LODDS to LNR and the pN classification, we therefore combined the pT stage and the two new N classifications (LODDS and rN) together to form the hypothetical TLM, TRM staging system and then compared them with the AJCC TNM staging system. The main finding of the present study is that the new TLM staging system is superior to the TRM or TNM staging system for prognostic prediction by using Cox regression multivariate analysis. Though the Kaplan-Meier plot shown a good discriminatory ability among stages IA through IIIC with all the three staging systems, we found that there was no significant difference between patients with stage IIA and IIB, P = 0.589. which was similar to our previous study [5]. The implementation of TLM staging system led to the identification of subgroups of patients prognostically more significantly than those classified by the TNM or TRM system. Though in the three multivariable regression analysis systems, TNM, TRM and TLM were all independent factors for the overall survival (P<0.001 for these three parameters. Table 5). We found that the −2log likelihood of the TLM staging system was the lowest and the HRs of the TLM staging system was the highest. Therefore we considered the TLM system was superior to the TRM and TNM system.

In our study, all the patients received D2 lymphadenectomy with R0 resection, and the majority of patients (78.4%) had more than 15 lymph nodes retrieved. Therefore we did not discuss the effect of lymph node number retrieved on the three staging systems.

The authors are not aware of any other studies addressing the superiority of TLM staging system in gastric cancer in China. In this investigation performed with 730 gastric adenocarcinoma we came to the following conslusions: 1) LODDS is superior to pN or rN classifications in predicting the 5-year overall survival rates of gastric adenocarcinoma patients. 2) The TLM staging system was better than the TRM or TNM.in predicting the overall survival of patients with gastric adenocarcinoma after D2 resection. Incorporating LODDS into the staging system of gastric cancer will enable clinicians to more accurately predict the prognosis of patients. The limitation of current study is in its retrospective analysis setting and from a single-institution experience. The impact of various treatments related outcome could not be evaluated fully in this study. External validation by using other large database for evaluating the prognostic effect of LODDS and TLM system must be taken prior to the recommendation for its practical usage.

## Methods

### Ethics Statement

All patients provided written informed consent for their information to be stored in the hospital database; we obtained separate consent for use of research. Study approval was obtained from independent ethics committees at Cancer Center of Sun Yat-Sen University. The study was undertaken in accordance with the ethical standards of the World Medical Association Declaration of Helsinki.

### Patients

Between January 1996 and January 2006, the medical records of 1000 pathology-proven gastric adenocarcinoma patients who were diagnosed and received treatment in the Cancer Center of Sun Yat-Sen University were retrospectively analyzed. Eligibility criteria were: (1) patients aged between 18 and 75 years of age, (2) patients receiving D2 resection carried out by experienced surgeons in our hospital following the Japanese Gastric Cancer Association (JGCA) guidelines [19], without macroscopic or microscopic residual tumor, (3) patients with a ≥3 months postoperative survival time and (4) patients without a history of other prior malignancy. Finally, 730 patients were included for the analysis.

The intervals of rN classification were determined by comparing overall survival rates according to rN with an initial interval of 0.1 and combining patients with similar prognosis (Table 2). Similarly, LODDS classification intervals were determined by comparing overall survival rates according to the value of LODDS with an initial interval of 0.5 and combing patients with similar prognosis (Table 3).

To make the study compatible with the 7^th^ edition of AJCC TNM staging system, we proposed another two staging systems on the basis of rN and LODDS classifications respectively. Considering that no patients with distant metastasis were included in this study, there is no stage IV patients in these three staging systems. The TRM staging system is as follows: IA, T1R0; IB, T1R1, T2R0; IIA, T1R2, T2R1, T3R0; IIB, T1R3, T2R2, T3R1, T4aR0; IIIA, T2R3, T3R2, T4aR1; IIIB, T3R3, T4aR2, T4bR0, T4bR1; IIIC, T4aR3, T4bR2, T4bR3. The TLM staging system is as follows: IA, T1L1; IB, T1L2, T2L1; IIA, T1L3, T2L2, T3L1; IIB, T1L4, T2L3, T3L2, T4aL1; IIIA, T2L4, T3L3, T4aL2; IIIB, T3L4, T4aL3, T4bL1, T4bL2; IIIC, T4aL4, T4bL3, T4bR4.

Clinical data collected for subsequent analysis included gender (male or female), age at diagnosis (<60 or ≥60. The median age was 60.), tumor size (≤5 cm or >5 cm), anemia (yes or no), primary tumor site (proximal or distal), degree of differentiation (well+moderate differentiated carcinoma or poor+signet ring cell differentiated carcinoma), total number of lymph nodes retrieved (<15 or ≥15), pT stage (7^th^ AJCC classification), pN stage (7^th^ AJCC classification), rN stage, LODDS stage, TNM stage (7^th^ AJCC classification), TRM stage and TLM stage (Table 1).

During the study period we did not have a standardized protocol for postoperative chemotherapy and (or) radiotherapy. Adjuvant therapy was suggested to all patients with T3–T4 classification or positive lymph node involvement; however, only 548 (75.1%) patients completed the adjuvant treatments. No patients received the adjuvant radiotherapy. Until July 2011, there were 321 patients died from the disease.

### Statistical analysis

All statistical analysis were performed by Statistical Package of Social Sciences 13.0 software. P value<0.05 was considered to be statistically significant. The Kaplan-Meier method was used to estimate the 5-year overall survival. For patients who remained alive, data were censored at the date of the last contact. Kaplan-Meier analysis with log-rank testing was used for univariate analysis. Overall survival rates were compared with different pN and rN classification when stratifying by LODDS and with different LODDS when stratifying by pN or rN classification. For the multivariate analysis, we firstly set up a model including age, status of anemia, size of tumor, tumor location, degree of differentiation, total number of lymph nodes retrieved and AJCC 7th TNM staging system. Then we set up a second model which was identical to the first one except that the AJCC 7th TNM staging system was replaced by the TRM staging system. In the third model we used the TLM staging system to replace the TRM system. We used two parameters to compare the TNM, TRM and TLM staging system, the −2log likelihood and the hazard ratio (HR). The higher the HR, the better the system. While the smaller the −2log likelihood, the better the system.
